# Correction: Arterial smooth muscle cell PKD2 (TRPP1) channels regulate systemic blood pressure

**DOI:** 10.7554/eLife.60403

**Published:** 2020-06-30

**Authors:** Simon Bulley, Carlos Fernández-Peña, Raquibul Hasan, M Dennis Leo, Padmapriya Muralidharan, Charles E MacKay, Kirk W Evanson, Luiz Moreira-Junior, Alejandro Mata-Daboin, Sarah K Burris, Qian Wang, Korah P Kuruvilla, Jonathan H Jaggar

Bulley S, Fernández-Peña C, Hasan R, Leo MD, Muralidharan P, Mackay CE, Evanson KW, Moreira-Junior L, Mata-Daboin A, Burris SK, Wang Q, Kuruvilla KP, Jaggar JH. 2018. Arterial smooth muscle cell PKD2 (TRPP1) channels regulate systemic blood pressure. *eLife* **7**:e42628. doi: 10.7554/eLife.42628.

Published 4, December 2018

It has come to our attention that we made a typographical error in reporting catalog numbers in this article related to the anti-PKD2 antibodies. The mouse monoclonal anti-PKD2 primary antibody from Santa Cruz was originally listed incorrectly as catalog number sc-100415. We in fact used two different catalog numbers for the mouse monoclonal anti-PKD2 primary antibodies from Santa Cruz. The *correct* catalog numbers for these antibodies are sc-28331 and sc-47734.

The article has been corrected accordingly. We apologize for the error.

