# Supplementary material for: Arterial smooth muscle cell PKD2 (TRPP1) channels regulate systemic blood pressure
Source: eLife. 2018 Dec 4;7:e42628. doi: 10.7554/eLife.42628 (PMC6281320; doi:10.7554/eLife.42628)
Supplement: Supplementary file 1. — The PKD2 forward primer recognizes nucleotides in exon 9 and 10 and the reverse primer was aligned with a sequence in exon 13. [file elife-42628-supp1.docx]

| **Transcript** | **Primer Forward** | **Primer Reverse** |
| --- | --- | --- |
| PKD2 | 5'-TGGGGCCACTTTATTTTACTACATTTG-3' | 5'-ATCGGACGTGGTAAAGAGCTGTGT-3' |
| AQP4 | 5′-GATCCTCTACCTGGTCACA-3′ | 5′-CACAGCTGGCAAAAATGGTA-3′ |
| Myh11 | 5′-AGGAACTGGAGGCGCTCAAGACA-3′ | 5′-TTGTCACTTCCTGTTCCCTC-3′ |
| PECAM1 | 5′-TCTTTCAGGATTCAGCTGAG-3′ | 5′-GCCGACTTTCCATATGGATG-3′ |
| Actin | 5′-GTGACGTTGACATCCGTAAAGA-3′ | 5′-GCCGGACTCATCGTACTCC-3′ |
